# Ultrasound dilution cardiac output and echocardiography findings in anesthetized mature alpacas (*Vicugna pacos*) during normotension, hypotension and hypertension

**DOI:** 10.1371/journal.pone.0284299

**Published:** 2023-04-10

**Authors:** Noelia Diaz-Falcon, Stuart Clark-Price, Merrilee Holland, Jacob Johnson, Kara Lascola

**Affiliations:** Department of Clinical Sciences, College of Veterinary Medicine, Auburn University, Auburn, Alabama, United States of America; Cairo University Kasr Alainy Faculty of Medicine, EGYPT

## Abstract

Alpacas (*Vicugna pacos*) have physiologic adaptations to live at high altitude. These adaptations may result in unexpected responses to changes in cardiac performance and blood pressure during general anesthesia. There are few studies evaluating cardiovascular variables in anesthetized alpacas. The purpose of this study was to report cardiovascular performance in anesthetized mature alpacas during normotension, hypotension, and hypertension using ultrasound dilution and echocardiography. Six adult alpacas, 3 females and 3 castrated males, weighing 62.6 to 88.7 kg were anesthetized and maintained with isoflurane and placed in right lateral recumbency. Each alpaca underwent ultrasound dilution and echocardiography measurements during three cardiovascular phases, normotension, hypotension via increased isoflurane concentration, and hypertension via phenylephrine infusion. Variables were analyzed with a Friedman test and a post hoc Dunn’s test when significant. A *p* < 0.05 was used for significance. Cardiac output, cardiac index, systemic vascular resistance, stroke volume, total ejection fraction, left ventricular internal diameter during diastole, and total stroke volume indexed to body weight were greater for hypertension compared to hypotension. Total ejection fraction, stroke volume, and left ventricular ejection time were greater for hypertions compared to normotension. There was no difference between ultrasound dilution and echocardiography determined cardiac output measurements within each cardiovascular phase. Phenylephrine appeared to have increased ventricular performance and/or increased preload in anesthetized, mature alpacas. For detecting change in cardiovascular status in anesthetized alpacas, ultrasound dilution and echocardiography may be useful.

## Introduction

Alpacas (*Vicugna paco*) are commonly domesticated pack and fiber animals and, more recently have gained popularity as companion animals [1]. As one of four species of South American camelids, alpacas have evolved in hypobaric and hypoxic conditions of high altitude. Thus, alpacas have developed genetic cardiovascular adaptations such as high blood oxygen affinity and pronounced peripheral vasoconstrictor responses [2,3]. It is not well understood if these adaptations may impact cardiac performance and blood pressure during general anesthesia of alpacas. Currently there are few studies evaluating cardiovascular variables in anesthetized alpacas. Dobutamine and norepinephrine increase cardiac index and blood pressure with dobutamine acting primarily via β1-adrenergic receptor stimulation and norepinephrine acting primarily via α-adrenergic receptor stimulation [4]. Controlled studies evaluating hemodynamic variables in anesthetized alpacas with various blood pressure states is warranted to provide improved anesthetic care.

Cardiac output (CO), the volume of blood pumped by the heart per unit of time, is a cardiac performance variable used in research studies evaluating anesthetized animals. The practical gold standard method to measure CO uses pulmonary arterial catheters (PAC) introduced into the heart and has been associated with complications such as pneumothorax, arrhythmias, pulmonary artery rupture, valve injury, and embolism in humans [5,6]. Thus, minimally invasive, or non-invasive methods have been introduced.

Ultrasound velocity dilution (UDCO) is a relatively new technique for determining CO and other cardiovascular variables that eliminates many of the disadvantages of PAC use. It utilizes peripherally placed catheters, does not involve blood loss, and uses a physiologic non-cumulative signal (isotonic fluid) [7,8]. The technique follows the theory of ultrasound velocity dilution, which is based on changes in blood ultrasound velocity through transient changes in blood protein concentration [9].

Transthoracic echocardiography (ECHO) can also be used to assess cardiac anatomy and function in a non-invasive manner. Multiple approaches have been proposed to calculate CO using ECHO in humans [10]. Following a volumetric method, calculating the difference of end-systolic and end-diastolic volume, it is possible to measure stroke volume (SV) and CO [11]. This can be achieved with M-mode (motion-based) ultrasonographic imaging. M-mode has been the leading echocardiography modality for approximately 10 years in human medicine until 2D and Doppler techniques were developed [12]. This approach has been used to measure CO in people, small animals, and adult horses [13–15]. To date, there is no published information on the use of UDCO to evaluate CO in alpacas. Information on the use of ECHO in alpacas is limited to case descriptions of cardiac diseases [16,17].

Phenylephrine is a selective α1-adrenergic receptor agonist that has been used to induce vasoconstriction and increased blood pressure for evaluation of blood pressure and other cardiac performance measurement devices [18,19].

The purpose of this study was to report cardiovascular performance in anesthetized mature alpacas during normotension (NORMO), hypotension (HYPO) induced with isoflurane, and hypertension (HYPER) induced with phenylephrine using UDCO and ECHO. The hypotheses were: 1) when compared to the normotensive state (baseline), CO would decrease during treatment HYPO induced with inhalant anesthetic; 2) CO would decrease during treatment HYPER induced with phenylephrine; 3) CO measurement via UDCO for each treatment would not be different from CO measurement via M-mode ECHO.

## Materials and methods

### Animals

Six adult alpacas, 3 females and 3 castrated males were used in the study. They were part of the university teaching hospital herd and were acclimated to being handled. Age ranged from 9 to 17 years old and body weight ranged from 62.6 to 88.7 kg. Alpacas were determined to be healthy based on normal findings on clinical examination and complete blood count and serum chemistry panel prior to the study. The study was approved by Auburn University Institutional Animal Care and Use Committee (protocol #2019–3523). At the conclusion of the study, all alpacas were returned to the teaching herd.

### General anesthesia and instrumentation

Food but not water was withheld for 12 hours prior to administration of any medications. The day before general anesthesia, alpacas were acclimated in pairs for 24 hours in a stall in a dedicated food animal medical barn to minimize stress. On the day of the study, a 14-gauge 5.5-inch polyurethane catheter (Surflo 14G X 5.5”I.V. catheter, Terumo, NJ, USA) was aseptically placed in the left jugular vein under minimal physical restraint. Prior to insertion of the catheter, the thoracic inlet was identified via manual palpation and the insertion point into the jugular vein was determined so that the tip of the catheter would be at least two inches into the thoracic cavity (approximately 3 inches cranial to the thoracic inlet). Anesthesia was induced with propofol (Propoflo; Zoetis, Kalamazoo, MI, USA) at 6 mg/kg IV to effect. Animals were placed in sternal recumbency for orotracheal intubation with a 12 mm internal diameter cuffed orotracheal tube. Animals were then positioned in right lateral recumbency and the endotracheal tube was attached to a rebreathing circuit attached to an anesthesia machine (Tafonius Jr., Hallowell EMC, MA, USA). Maintenance of anesthesia was performed with isoflurane (Isoflurane, Akorn, Inc., Lake Forest, IL, USA) delivered in oxygen at 4 L per minute. Alpacas’ lungs were mechanically ventilated with a tidal volume (V_T_) of 15 ml/kg and a rate of 8 breaths per minute to maintain end tidal CO_2_ between 34–45 mm Hg. Alpacas were instrumented with a dedicated patient monitor (Mindray Passport 12, Mindray North America, NJ, USA) and electrocardiogram, pulse oximetry, and end-tidal gas analysis were monitored. A 20-gauge, 2-inch catheter (Surflash 20G x 2”, Terumo, NJ, USA) was placed in a caudal branch of the right femoral artery at the midpoint of the medial aspect of the thigh just caudal to the femur. Three way stop-cocks were placed on the arterial and jugular catheters and pressure transducers (TruWave Pressure Transducer, Edwards Lifesciences, CA, USA) were attached to one limb of each stopcock with non-compliant, 36 inch, saline filled tubing manufactured specifically for pressure monitoring (36in Monitoring Line, Smith Medical, OH, USA). The pressure transducers were connected to the patient monitor, zeroed to atmospheric pressure, and positioned at the level of the manubrium to approximate the right atrium to measure arterial blood pressure and central venous pressure. New pressure transducers were used for each alpaca and were guaranteed by the manufacturer to have an accuracy of ± 1 mmHg plus 1% of reading from -50 to +50 mmHg and ± 2.5% of reading from +50 to +300 mmHg (package insert, TruWave Pressure Transducer, Edwards Lifesciences, CA, USA). Vital variables and end tidal concentration of isoflurane were recorded at five minutes intervals during general anesthesia. The gas analyzer component of the patient monitor was checked for accuracy at weekly intervals against a standard calibration gas per the manufacturer’s recommendation (calibration gas, Airgas Healthcare, PA, USA). Mechanical ventilation was kept constant throughout the data collection period.

### UDCO measurement

Ultrasound dilution cardiac output measurements were performed with the COstatus System device (Transonic Systems, Ithaca, NY). For each alpaca, the arterial catheter was connected with the central jugular catheter through an extracorporeal arteriovenous (AV) loop. This AV loop was connected to a peristaltic pump that kept blood flow constant during measurements. Two ultrasound velocity sensors were attached to this circuit, a venous sensor was placed just upstream from the venous catheter and the arterial sensor just downstream from the arterial catheter. The sensor on the venous side measured the amount of the injected volume and the quality of the injection. The sensor on the arterial side measured a dilution curve. The dilution curve was created by a change in protein concentration of blood after injection of an isotonic saline indicator bolus as it circulates through the cardiovascular system [9]. The bolus consisted of 30 ml of warmed isotonic saline rapidly injected into the venous side of the AV loop. Cardiac output was determined with a variation of the Stewart-Hamilton principle:

$$CO=\frac{\left(UVblood-UVsaline)*(Vinj\right)}{\int UVa(t)dt}=\frac{Vinj}{\int Ca(t)dt}[L/min]$$

Where *Vblood−UVsaline* is the difference between the ultrasound velocities in blood and saline measured by the venous sensor, *Vinj* is the volume of isotonic saline injected (ml) measured by the venous sensor, *UVa* is the change in arterial blood ultrasound velocity measured by the arterial sensor; *Ca*(*t*) is the concentration of injected saline in arterial blood, and ∫*Ca*(*t*)*dt* is the area under the dilution curve of the saline concentration in arterial blood [20,21].

Other Hemodynamic variables determined by calculation were cardiac index (CI), systemic vascular resistance (SVR), stroke volume (SV), and total ejection fraction (TEF). Cardiac index was calculated as CO normalized to body weight (kg^0.67^):

$$CI=\frac{CO}{{kg}^{0.67}}\;[mL/min/{kg}^{0.67}]$$

Systemic vascular resistance was calculated as:

$$SVR=(MAP-CVP)*\frac{80}{CO}\;[dynes*sec*{cm}^{-5}]$$

Where *MAP*−*CVP* is the difference between mean arterial pressure (MAP) and central venous pressure (CVP).

Stroke volume was calculated as:

$$SV=\frac{CO}{HR}\;[mL/beat]$$

Where HR is heart rate.

Total ejection fraction was calculated as:

$$TEF=\left(\frac{4*SV}{TEDV}\right)*100\;[\%]$$

Where SV is stroke volume and TEDV is total end diastolic volume and the number 4 is used to account for all chambers of the heart.

### Transthoracic echocardiography measurements

Transthoracic echocardiography was performed with a GE Vivid E9 ultrasound machine with a phase array transducer (GE healthcare, Fairfield, CT). A right thoracic window was used to obtain a two-dimensional view (2-D) of the short axis of the left ventricle (LV) with M-mode guidance. The specific imaging plane used for measurements was based on what the examiner regarded as the most accurate and representative view of the LV. From M-Mode, a LV internal diameter at end-diastole (LVIDd) and at end-systole (LVIDs) was measured and used to determine the left ventricular volume at systole and diastole. Total stroke volume indexed to body weight (TOT SV:BW) was derived from the difference of left ventricle (LV) end-diastolic and end-systolic volumes calculated respectively as LVEDD^3^ and LVESD^3^. Cardiac output was calculated as the product of stroke volume (SV) and heart rate (HR). In addition, the interventricular septum at end-diastole (IVSd) and end-systole (IVSs) and LV free wall thickness at end-diastole (LVFWd) and end-systole (LVFWs) were measured, obtained from a right short axis view during systole and diastole. Fractional shortening (FS), expressed as percentage, was also obtained for each blood pressure treatment. The left ventricular ejection time (LVET) was measured from the spectral tracing of the aortic valve.

### Experimental design

Each alpaca underwent UDCO and ECHO measurements during three cardiovascular phases, treatment NORMO, treatment HYPO, and treatment HYPER. During each phase, measurement with UDCO was performed simultaneously with ECHO. Each measure was taken in triplicate and averaged for each time point. The ECHO examinations were performed by the same board-certified veterinary radiologist (MH) with extensive experience with ECHO. Prior to the first measurements, alpacas were allowed a period of at least 15 minutes to achieve anesthetic equilibration (inspiratory isoflurane concentration equal to expiratory isoflurane concentration) that resulted in a steady mean arterial blood pressure between 65 and 75 mm Hg (NORMO). After achieving normotension, UDCO and ECHO were performed for treatment NORMO. Next, hypotension was induced by increasing the inspired concentration of isoflurane until MAP was between 45 and 55 mmHg. Once the desired hypotension was achieved, the vaporizer setting was maintained for a period of at least 15 minutes to allow for anesthetic equilibration and UDCO and ECHO were performed for treatment HYPO. The vaporizer setting was not altered until after all measurement were performed. Finally, the isoflurane concentration was returned to baseline and an IV phenylephrine infusion was initiated for treatment HYPER. The initial phenylephrine infusion rate was 0.5 mcg/kg/min for the first five minutes. Afterwards, the rate was increased by 0.5 mcg increments at two minute intervals until a MAP greater than 85 mmHg was achieved. The infusion was maintained for a minimum of 15 minutes was allowed for anesthetic and phenylephrine equilibration and then UDCO and ECHO were performed. Phenylephrine and isoflurane administration were then discontinued and alpacas were allowed to recover from anesthesia.

### Statistical methods

A sample size calculation was performed to detect a difference of 1 L/min in CO between blood pressure states with a sigma of 0.58 [22], an alpha of 0.05 and a power of 0.8 indicating the need of 6 animals. One L/min in the sample size calculation was used as it was considered to be a clinically relevant change in CO. Descriptive statistics for each measured variable were calculated and reported as median (range). Due to a small sample size, all data were subsequently analyzed with non-parametric tests to reduce the chance of Type 1 error. Variables were analyzed with a Friedman test and a *post hoc* Dunn’s test when significant. A *p* value < 0.05 was used for significance.

Sample size calculation was performed with an online sample size calculator (University of California San Francisco Biostatistics: Power and Sample Size Program, https://www.stat.ubc.ca/~rollin/stats/ssize/). All other statistics were performed with a commercially available statistics program (InStat version 3.10, GraphPad Software, CA, USA).

## Results

All 6 alpacas completed the study successfully. No complications during general anesthesia or recovery were noted. Descriptive data for HR, SAP, MAP, and DAP are summarized in Table 1. Heart rate was not significantly different among treatments. Systolic, mean, and diastolic pressures were different among treatments. The median (range) end-tidal isoflurane (%) for each blood pressure treatment was 2.05 (1.35–2.42) for HYPO, 1.22 (0.8–1.63) for NORMO, and 1.39 (1.05–1.62) for HYPER. The median (range) dosage for phenylephrine for treatment HYPER was 1 (0.5–3) mcg/kg/min.

**Table 1:** Median (min-max) values for heart rate and arterial blood pressure in six, isoflurane anesthetized, mature alpacas during isoflurane induced hypotension, normotension and phenylephrine infusion (0.5–3 mcg/kg/min) induced hypertension. Hypotension was defined as a mean blood pressure between 45 and 55 mmHg, normotension was defined as a mean blood pressure between 65 and 75 mm Hg and hypertension was defined as a mean blood pressure greater than 85 mmHg.

	Blood Pressure Status		
Variable	Hypotension	Normotension	Hypertension
HR (beats/min)	76 (60–86)	76 (66–110)	78 (55–114)
Systolic (mmHg)	66 (63–77)^#^	100 (90–122)^§^	159 (139–192)*
Mean (mmHg)	47 (39–52)^#^	70 (61–82)^§^	117 (110–140)*
Diastolic (mmHg)	37 (30–42)^#^	59 (47–78)^§^	103 (88–124)*

*Significant difference between hypotension and hypertension (*p < 0*.*001*).

^§^Significant difference between normotension and hypertension (*p < 0*.*05*).

^#^Significant difference between hypotension and normotension (*p < 0*.*05*).

### Ultrasound dilution cardiac output

Results for CO, CI, SVRI, SVI, and TEF obtained via UDCO for each blood pressure treatment are summarized in Table 2. Cardiac output, CI, SVRI, SVI, and TEF were significantly higher for HYPER than for HYPO (all *p* < 0.05). Cardiac output increased 48%, CI increased 73%, SVRI increased 61%, and TEF increased 33% from HYPO to HYPER. There were no significant difference in CO, CI, SVRI, SVI, or TEF between HYPER and NORMO or between NORMO and HYPO.

**Table 2:** Median (min-max) values for cardiac variables measure with UDCO in six, isoflurane anesthetized, mature alpacas during isoflurane induced hypotension, normotension and phenylephrine infusion (0.5–3 mcg/kg/min) induced hypertension. Hypotension was defined as a mean blood pressure between 45 and 55 mmHg, normotension was defined as a mean blood pressure between 65 and 75 mm Hg and hypertension was defined as a mean blood pressure greater than 85 mmHg.

	Blood Pressure Status		
Variable	Hypotension	Normotension	Hypertension
CO (L/min)	4.46 (2.14–4.63)	4.3 (3.03–5.74)	6.62 (4.22–8.90)*
CI (L/min/m^2^)	3.8 (2.01–5.42)	8.83 (2.84–6.68)	6.58 (3.52–10.04)*
SVRI (dynes*sec*cm^-5^/m^2^)	808 (563–1447)	1370 (690–1983)	1303 (903–3120)*
SVI (mL/m^2^)	54 (35–71)	50 (34–92)	72 (60–121)*^§^
TEF (%)	40 (34–45)	44 (37–48)	53 (42–53)*^§^

*Significant difference between hypotension and hypertension (*p < 0*.*05*).

^§^ Significant difference between normotension and hypertension (*p < 0*.*05*).

CO = cardiac output, CI = cardiac index, SVRI = systemic vascular resistance index, SVI = stroke volume index, TEF = total ejection fraction.

### Transthoracic echocardiography

Results for CO, IVSd, IVSs, LVIDd, LVIDs, LVFWd, LVFWs, FS, LVET, and TOT SV:BW obtained via ECHO for each blood pressure treatment are summarized in Table 3. Cardiac output, LVIDd, TOT SV:BW were significantly higher for HYPER than for HYPO (*p* < 0.05). Cardiac output increased 118%, LVIDd increased 22%, and TOT SV:BS increased 156%. There was no significant difference in CO between HYPER and NORMO or between NORMO and HYPO. There was no significant differences in LVIDd between HYPER and NORMO or between NORMO and HYPO. Left ventricular ejection time was significantly longer (34%) for HYPER compared to NORMO (*p* < 0.05). There were no significant differences in LVET between HYPER and HYPO or NORMO and HYPO. There were no significant differences in TOT SV:BW between HYPER and NORMO or between NORMO and HYPO. There was no significant difference among treatments for IVSd, IVSs, LVIDs, LVFWd, LVFWs, or FS.

**Table 3:** Median (min-max) values for hemodynamic variables measured with ECHO in six, isoflurane anesthetized, mature alpacas during isoflurane induced hypotension, normotension and phenylephrine infusion (0.5–3 mcg/kg/min) induced hypertension. Hypotension was defined as a mean blood pressure between 45 and 55 mmHg, normotension was defined as a mean blood pressure between 65 and 75 mm Hg and hypertension was defined as a mean blood pressure greater than 85 mmHg.

	Blood Pressure Status		
Variable	Hypotension	Normotension	Hypertension
CO (L/min)	2.66 (2.21–4.23)	5.01 (2.85–7.25)	5.79 (4.48–15.89)*
IVSd (cm)	1.30 (1.16–1.49)	1.26 (1.13–1.50)	1.21 (1.09–1.41)
LVIDd (cm)	4.06 (3.91–4.41)	4.72 (4.24–5.08)	4.94 (4.69–5.43)*
LVIDs (cm)	3.13 (2.88–3.36)	3.52 (3.08–3.95)	3.43 (2.93–3.68)
LVFWd (cm)	1.16 (0.99–1.71)	1.20 (1.01–1.38)	1.06 (0.95–1.17)
FS (%)	25 (19–37)	26 (16–31)	33 (26–40)
LVET (sec)	0.29 (0.24–0.38)	0.29 (0.25–0.34)	0.39 (0.37–0.41)§
TOT SV:BW (mL/kg)	0.50 (0.40–0.62)	0.79 (0.46–1.16)	1.28 (0.71–1.39)*

*Significant difference between hypotension and hypertension (*p < 0*.*05*).

^§^ Significant different between normotension and hypertension (*p < 0*.*05*).

CO = cardiac output, IVSd = interventricular septum thickness at end-diastole, LVIDd = left ventricular internal diameter at end-diastole, LVIDs = left ventricular internal diameter at end- systole, LVFWd = left ventricular free wall thickness at end diastole, FS = fractional shortening, LVET = left ventricular ejection time, TOT SV:BW = total stroke volume indexes to body weight.

### Comparison of CO measurement between methods

Results for comparison of CO measurements between UDCO and ECHO for each treatment are summarized in Table 4. There was no statistical difference between UDCO and ECHO CO measurements within each treatment.

**Table 4:** Comparison of median (min-max) values for cardiac output measured via ultrasound dilution or M-mode echocardiography in six, isoflurane anesthetized, mature alpacas during isoflurane induced hypotension, normotension and phenylephrine infusion (0.5–3 mcg/kg/min) induced hypertension. Hypotension was defined as a mean blood pressure between 45 and 55 mmHg, normotension was defined as a mean blood pressure between 65 and 75 mm Hg and hypertension was defined as a mean blood pressure greater than 85 mmHg.

	CO (L/min)		
Blood Pressure State	UDCO	ECHO	p value
Hypotension	4.46 (2.14–4.63)	2.66 (2.21–4.23)	>0.05
Normotension	4.3 (3.03–5.74)	5.01 (2.85–7.25)	>0.05
Hypertension	6.62 (4.22–8.90)	5.79 (4.48–15.89)	>0.05

CO = cardiac output, UDCO = ultrasound dilution cardiac output, ECHO = M-mode echocardiography.

## Discussion

To the authors’ knowledge, this is the first report of CO and other hemodynamic variables measured with UDCO and ECHO in mature, anesthetized alpacas during different blood pressure states. Contrary to our hypothesis, when compared to treatment NORMO, neither HYPO induced by isoflurane nor HYPER induced by phenylephrine resulted in significant reductions in CO when measured by either UDCO or ECHO. Cardiac output measurement with UDCO and ECHO were not significantly different between modalities within treatments.

Phenylephrine was chosen to be used as a means to increase blood pressure for evaluation of UDCO and ECHO performance in this study because its use for manipulation of blood pressure for evaluation of cardiovascular measurement devices in other species [18,19,23–25].

Cardiac output can be decreased by an increase in SVR through impedance of ejection of blood or the force that resists ventricular muscle contraction [26]. This phenomenon has been demonstrated with the use of phenylephrine in anesthetized dogs and horses and is thought to be repeatable in most mammalian species [27,28]. It was therefore an unexpected finding that CO increased in the alpacas in the treatment HYPER in this study. Reasons for the increased CO could be the activation of myocardial alpha-1 adrenergic receptors by phenylephrine or an increase in venous return to the heart from vascular constriction.

Alpha adrenergic receptors are mainly located within pre and postsynaptic regions of the sympathetic nerve endings on smooth muscle cells [29]. However, from cellular cardiology studies performed in humans and in laboratory animals, there are also smaller but functionally important populations of beta-2 and alpha-1 myocardial adrenergic receptors [30]. In cats, a phenylephrine infusion (2 mcg/kg/min), increases cardiac index (CI) with an increased stroke volume index (SVI), demonstrating a possible positive inotropic action, although the increased SVI could have been related to increased venous return [31]. In laboratory animals, activation of alpha-1 adrenergic receptors induces a positive inotropic response in left ventricular myocytes [32–34]. Additionally, in humans, stimulation of myocardial alpha-adrenergic receptors results in similar positive ionotropic effect [35]. Large concentrations of alpha-1 receptors have been identified in the myocardium of humans, mice, guinea pig, rabbit, pigs and cows and likely play a role in cardiac performance [36].

A second explanation for the increase in CO could be due to an increase in preload secondary to phenylephrine induced constriction of capillaries, peripheral veins, and central veins with a subsequent increase in stroke volume [37]. In a study in pigs that were experimentally treated to be adjusted on the Frank-Starling curve to make CO preload independent or preload dependent, phenylephrine increased CO in pigs on the preload dependent part of the curve but decreased CO in pigs on the preload independent part of the curve. This indicates that the effect of phenylephrine on CO is related to preload dependency and may be useful in animal in need of increased preload [38]. Of interest, newborn llamas have a higher peripheral vascular reactivity in response to alpha-1 stimulation than lowland species due to preferential expression of an alpha-1B receptor subtype [39,40]. This represents an adaptation that allows for an ability to redistribute blood in the face of a hypoxic (high altitude) environment. Although unknown, it is possible that alpacas carry a similar alpha receptor subtype densities in the vasculature as they originate from a similar high altitude environment.

It could be argued that the increase in CO seen in treatment HYPER compared to treatment HYPO was due to decreased concentration of isoflurane delivered to treatment HYPER. The isoflurane concentration in treatment HYPER was similar to treatment NORMO to return MAP to treatment NORMO values prior to initiation of the phenylephrine infusion. Isoflurane tends to have a dose dependent depressive effect on cardiovascular function. For example in dogs, CO decreases from a decrease in SV and SVR during isoflurane anesthesia [41]. However, increased dosage of isoflurane in anesthetized adult llamas does not result in a change in SV, CI, or total peripheral resistance similar to the finding in alpacas in this study between treatment HYPO and treatment NORMO [42]. These findings suggest that the increase in CO in treatment HYPER was not solely due to change in isoflurane concentration alone. However, the effect of the change in isoflurane concentration from treatment HYPO to treatment HYPER could not be determined as cardiac measurements were not repeated at return to normotensive MAP prior to initiation of phenylephrine infusion.

Similar to CO, several other cardiac variables determined with UDCO increased in treatment HYPER compared to treatment HYPO. Cardiac index is simply CO normalized to body weight, and since CO increased in treatment HYPER compared to treatment HYPO, it would be expected that CI would be increased in treatment HYPER as well. An expected result was the increase in SVR in treatment HYPER compared to treatment HYPO. Phenylephrine, which causes a dose-dependent increase in SVR, was utilized in this study specifically to increase vascular tone and SVR in order to evaluate the effects of hypertension on CO. Stroke volume, the volume of blood pumped from the left ventricle during a single contraction, is one of the determinants of CO. Stroke volume was increased in treatment HYPER compared to treatment HYPO potentially indicating a positive inotropic effect from the administered phenylephrine. Total ejection fraction is the fraction of blood of the end-diastolic volume in the left ventricle ejected during contraction. This value, calculated by UDCO, is used as a measure of contractile strength of the ventricular myocardium. In treatment HYPER, TEF increased compared to treatments HYPO and NORMO again suggesting a positive inotropic effect from phenylephrine administration. Measured via ECHO, LVIDd increased in treatment HYPER compared to treatment HYPO. This finding supports the thought that phenylephrine induced vascular constriction results in a greater preload and thus an increase in CO. Left ventricular ejection time is the time interval from the aortic valve opening to aortic valve closure and directly correlated with SV. In this study LVET increased in treatment HYPER compared to treatments HYPO and NORMO and should be expected with the increase in SV resulting in the increase in CO. Finally, TOT SV:BW was increased in treatment HYPER compared to treatment HYPO. This is an expected finding given that SV and CO increased in that treatment as well.

An additional outcome of this study was the comparison of CO measurement by UDCO and ECHO. Statistically, the measurements between the two methods were found not to be different within the treatment groups. However, further comparison should be made with greater numbers of animals to assess bias and limits of agreement. This study was likely underpowered to make a definitive statement on the agreement of UDCO with ECHO. However, the usefulness of the measurement methods of UDCO and ECHO for CO measurement is further supported by a systematic review and meta-analysis in humans that found CO measurement by ECHO or pulmonary artery catheterization were not different [43]. Furthermore, M-mode ECHO has been found to be accurate and useful for rapid, noninvasive estimation of CI in dogs without clinically apparent heart diseases [44].

The main limitation of this study was the use of methods of CO measurement that have not been validated in alpacas. The practical gold standard method for measurement of cardiac output is thermodilution via pulmonary artery catheterization. However, this method has been associated with serious adverse events including pulmonary thrombosis, arrhythmias, and pulmonary artery rupture in humans [45]. There have not been any reports of complications associated with pulmonary artery catheterization in alpacas. The use of UDCO and ECHO represent minimally or non-invasive methods for CO measurement. The alpacas used in this study were not available for placement of a pulmonary arterial catheter and thus UDCO and ECHO were utilized even though they have not been validated for use in this species. The UDCO method has been validated in adult, pediatric and neonatal humans, sheep, dogs, neonatal and juvenile horses, and pigs [46–52]. Thus, it is likely that the results obtained in this study are accurate. Furthermore, in a previous study in alpacas that did utilize pulmonary artery catheters for CO measurement to assess the effects of butorphanol on cardiovascular variables, the values obtained for the control treatment were nearly identical to the values obtained from treatment NORMO in this study further supporting the use of UDCO in alpacas [22].

Another limitation of this study included a small sample size (6 alpacas) which may have affected the results, particularly for comparisons between treatments HYPO and NORMO. While the median values for CO between HYPO and NORMO treatments were similar, treatment NORMO was not statistically different from treatment HYPER even though treatments HYPO and HYPER were different. The lack of difference between treatments NORMO and HYPER was likely due to the wide variability among animals suggesting the number of animals used in the study may not have provided enough power. Enrolling additional alpacas in the study may have resulted in a statistical difference between treatments NORMO and HYPER. Additionally, there were no intact male alpacas used in this project. This was because the alpacas that were available for this study are part of a teaching herd and are housed together. Intact males are not permitted to prevent breeding and to minimize aggressive behavior. It is not known if the use of intact male alpacas would have impacted the results of this study.

The use of intermittent positive pressure ventilation (IPPV) could have interfered with the CO measurements. During IPPV, elevated intrathoracic pressure is transmitted against the right atrial wall, reducing venous return. The reduction in right-sided preload, causes a reduction in right-sided cardiac output [53]. Alpacas in this study were mechanical ventilated using a tidal volume (TV) similar to normal awake standing (12.13 ± 2.54 mL/kg) or sternally recumbent (14.02 ± 3.10 mL/kg) alpacas [54]. This likely resulted in minimal impact on CO as there were no appreciable variations in arterial pulse waves.

Finally, the order of the hemodynamic states during measurements was not randomized. This was done due to lack of information regarding the effect and duration of action of phenylephrine in alpacas. Concern was that if there was a prolonged effect from the phenylephrine, the alpacas would have needed to be maintained under anesthesia for an unacceptably long period of time. Therefore, the induced hypertension was the last state so as not to potentially interfere with the NORMO or HYPO treatments.

Future studies in alpacas are warranted to support the use of UDCO and ECHO for monitoring hemodynamic variables. Additionally, investigation into the mechanism behind the increase in CO associated with phenylephrine administration as well as the effects of other inotropic or vasopressor agents would be of high clinical interest for the management of alpacas during anesthesia.

In conclusion, in isoflurane anesthetized, healthy, mature alpacas, isoflurane induced hypotension can result from a decreasing in CO and other cardiovascular variables. In hypotensive isoflurane anesthetized, mature alpacas, a phenylephrine infusion along with reduction in isoflurane concentration may increase in CO, and other cardiovascular variables via increased ventricular performance and/or increased preload. Additionally, the results of this study, suggest that UDCO and ECHO may be viable methods for detecting changes in cardiovascular variables in anesthetized alpacas.

## Supporting information

S1 Dataset Main data set. (XLSX)
